# Perioperative nutrition-related laboratory profiles predict outcomes after digestive procedures in patients with gastrointestinal cancer

**DOI:** 10.3389/fnut.2026.1927720

**Published:** 2026-10-15

**Authors:** Menghui Zhang, Liangliang Hui, Jialing Zhang, Chengran Wang, Fan Xu, Guoquan Tao, Honggang Wang

**Affiliations:** 1 Department of Gastroenterology, The Affiliated Huaian No. 1 People’s Hospital of Nanjing Medical University, Huai'an, China; 2 Department of Intensive Care Unit, The Affiliated Huaian No. 1 People’s Hospital of Nanjing Medical University, Huai'an, China; 3 Department of Gastrointestinal Surgery, The Affiliated Huaian No. 1 People’s Hospital of Nanjing Medical University, Huai'an, China

**Keywords:** clinical nutrition, gastrointestinal cancer, INSPIRE, laboratory risk profile, MIMIC-IV, surgical outcomes

## Abstract

Routine laboratory measurements may capture nutritional, inflammatory, and organ-function vulnerability in patients with gastrointestinal cancer. We developed nested logistic regression models using INSPIRE v1.4.2 and evaluated a portable model in MIMIC-IV v3.1. The original INSPIRE cohort comprised 11,471 operations among 10,728 patients, with 893 deaths within 365 days. Patients were allocated to development and held-out test sets. For 365-day mortality, area under the receiver operating characteristic curve (AUROC) was 0.783 (95% CI 0.752–0.811) for clinical variables, 0.843 (95% CI 0.820–0.868) after adding laboratory variables, and 0.856 (95% CI 0.834–0.879) for the full perioperative model. Patient-level full-pipeline bootstrap analysis yielded an optimism-corrected AUROC of 0.837. External AUROC was 0.676 (95% CI 0.663–0.688) for 365-day mortality and 0.724 (95% CI 0.692–0.754) for recorded hospital death after harmonizing CRP units. Calibration drift was substantial, and hospital-death discrimination was sensitive to laboratory timing. Corrected operation-linked risk tertiles showed graded mortality and lower albumin and hemoglobin levels in higher-risk groups. Perioperative nutrition-related laboratory profiles provided additional discrimination for 365-day mortality beyond clinical variables, although limited external performance and calibration drift warrant further validation before clinical use.

## Introduction

Gastrointestinal cancers account for a substantial share of global cancer morbidity and mortality, and surgery remains central to curative-intent treatment for many patients (1). Yet, postoperative outcomes differ widely among patients undergoing apparently similar operations. Beyond tumor site and technical complexity, nutritional reserve, systemic inflammation, anemia, hepatic function, renal function, and coagulation status shape patients’ ability to tolerate major gastrointestinal surgery.

Clinical nutrition research has emphasized malnutrition as a potentially modifiable perioperative risk state. In an international cancer-surgery cohort, malnutrition was associated with adverse early surgical outcomes (2). Perioperative nutrition management and enhanced-recovery pathways are also considered part of gastrointestinal cancer surgery quality improvement (3, 4). Recent gastrointestinal oncology studies have linked albumin, prognostic nutritional indices, frailty, C-reactive protein-albumin composites, and other nutritional markers to complications or survival (5–9). These findings motivate the evaluation of nutrition-related laboratory measurements for risk prediction, while distinguishing their prognostic information from a direct assessment of nutritional status. In patients with gastrointestinal cancer, nutritional depletion may coexist with systemic inflammation, anemia, biliary obstruction, and impaired liver function. Broader perioperative prediction studies have combined clinical, physiologic, imaging, and electronic-health-record features for surgical risk stratification (10–16). A relevant question is how much a combined laboratory panel adds to clinical variables, including information beyond albumin and hemoglobin alone. Evaluation across datasets is also needed to assess whether this prognostic information translates into comparable discrimination and calibration in another setting.

Large perioperative electronic health record datasets allow these questions to be studied at scale. INSPIRE provides operation-level perioperative data, whereas MIMIC-IV provides a separate hospital dataset for evaluating a portable model across settings (17, 18). Using INSPIRE, we assessed whether routine nutrition-related laboratory measurements improved discrimination for 365-day mortality beyond clinical variables. The study population comprised patients with gastrointestinal cancer undergoing digestive procedures. We then evaluated the discrimination and calibration of a portable model in MIMIC-IV. We also described laboratory patterns across predicted-risk strata to characterize the resulting risk profiles. Prediction-model and routinely collected data reporting principles guided these analyses (19–21).

## Materials and methods

### Study design and data sources

We conducted a retrospective prediction-model development and external-validation study using de-identified public-use data. The primary cohort was derived from INSPIRE v1.4.2, a perioperative electronic health record dataset containing operation records, diagnosis codes, laboratory values, intraoperative vital signs, medication summaries, and outcome timestamps (17). External validation used MIMIC-IV v3.1, an independent hospital electronic health record dataset (18). Reporting was guided by TRIPOD+AI and RECORD (19, 21).

### Cohort definition

The operation was the analytic unit in INSPIRE. Eligibility required a recorded gastrointestinal malignancy diagnosis before or at operation start, an ICD-10-PCS prefix of 0D or 0F, and assignment to one of seven cancer groups; the unspecified digestive-cancer group was excluded. Diagnostic prefixes were C15 (esophageal), C16 (gastric), C17 (small intestine), C18–C21 (colorectal/anal), C22 (liver), C23–C24 (gallbladder/biliary), and C25 (pancreatic). The original category assignment used the patient-level diagnostic history and a fixed priority for multiple sites; a preoperative-only assignment was examined in a sensitivity analysis. These broad procedure codes do not establish curative resection, operative indication, or the absence of diagnostic procedures. Multiple operations were retained: 658 patients contributed more than 1 operation, accounting for 743 additional operations (maximum 5 per patient). Patients, rather than operations, were allocated in a 75:25 split using seed 20,260,702, yielding 8,587 development and 2,884 test operations with no patient overlap. MIMIC-IV included admissions with digestive malignancy and a qualifying procedure (ICD-10-PCS 0D/0F or ICD-9 procedure prefixes 42–54); the earliest qualifying procedure date per admission was used. Complete selection rules and reconciliation with the earlier secondary extraction are provided in Supplementary methods.

### Laboratory and perioperative predictors

Laboratory variables included albumin, bilirubin, aspartate aminotransferase (AST), alanine aminotransferase (ALT), alkaline phosphatase, prothrombin time international normalized ratio (PT-INR), platelet count, C-reactive protein (CRP), white blood cell count, hemoglobin, creatinine, lactate, glucose, HbA1c, blood urea nitrogen, sodium, and potassium. In INSPIRE, the closest non-missing measurement within 30 days before or at operation start was selected; narrower 7-day and 14-day windows were evaluated. Derived variables were the albumin–bilirubin (ALBI) score, albumin–bilirubin ratio, AST-to-platelet ratio index (APRI), and fibrosis-4 (FIB-4) index. Clinical variables were age, sex, body mass index, American Society of Anesthesiologists (ASA) class, emergency status, anesthesia type, cancer group, department, operation duration, and operating-room duration. The full model added intraoperative physiologic and treatment summaries. Durations and intraoperative measurements are not available at a preoperative decision point; these models therefore concern perioperative prediction. Raw laboratory measurements were not log-transformed, apart from the logarithm and square root transformations used within specified derived indices. Training-set medians and standardization were applied to numeric predictors, with categorical missingness coded as a distinct category and all categories one-hot encoded. Exact transformations are described in Supplementary methods. INSPIRE missingness is reported in Supplementary Table 2, and MIMIC-IV missingness in Supplementary Data. Numerical specifications of the original-cohort refitted models and comparisons with archived predictions are included in Supplementary Data.

### Outcomes and modeling

The primary outcome was recorded as all-cause death within 365 days after operation end in INSPIRE or from the qualifying procedure date in MIMIC-IV. The original secondary INSPIRE labels were non-missing source fields for ICU admission and hospital death; they were not independently adjudicated index-admission outcomes. Timing-restricted sensitivity analyses are reported separately. The original nested models used L2-penalized logistic regression (C = 1, liblinear solver, class_weight = balanced) rather than unpenalized regression. The portable models used the same default L2 penalty without class balancing. The primary full design contained 63 non-intercept columns and 660 development events (approximately 10.5 events per column); the previously reported 195 columns belonged to a different secondary reconstruction. Discrimination, average precision, Brier score, calibration, and exploratory decision curves were evaluated (22, 23). Confidence intervals were recomputed by 500 patient-level bootstrap samples, retaining all records of each sampled patient. For internal optimism assessment, the entire original development pipeline, including preprocessing, was refitted in 500 patient-level bootstrap samples. Additional analyses examined first operations, missingness indicators, narrower laboratory windows, preoperative-only cancer assignment, and an unweighted ridge model selected by five-fold patient-grouped cross-validation within the development set. These are internal sensitivity analyses of the original cohort, not independent external validation (Supplementary methods).

### External evaluation and descriptive risk profiles

The portable model used age, sex, cancer group, 17 laboratory measurements, and four derived indices. ASA, BMI, emergency status, department, anesthesia type, durations, and intraoperative variables were not transported because comparable definitions or measurements were unavailable in MIMIC-IV. The model was fitted only in INSPIRE. The original external extraction selected admission-linked laboratories through the end of the qualifying procedure date; those measurements cannot all be confirmed as preoperative. We harmonized MIMIC-IV CRP from mg/L to the INSPIRE mg/dL scale and repeated external evaluation using only results available before the procedure date. Calibration-in-the-large (slope fixed at one), joint recalibration of the intercept and slope, and observed-to-expected event ratios were calculated. A five-fold patient-grouped cross-fitted recalibration was evaluated within MIMIC-IV as a model updating procedure, but not as a new external validation. Risk tertiles were defined separately in each cohort from portable 365-day scores and linked one-to-one to operation records in INSPIRE and admission records in MIMIC-IV. Laboratory medians were centered and scaled using the corresponding cohort median and interquartile range. These descriptive strata are not common absolute-risk categories, independently established biological phenotypes, or treatment thresholds. Exploratory ICU comparisons use non-equivalent source definitions (Supplementary methods).

## Results

### Cohort characteristics and outcomes

The primary cohort remained 11,471 operations among 10,728 patients, with 893 recorded deaths within 365 days (7.8%). The largest cancer groups were colorectal/anal (4,413), gastric (3,906), liver (1,445), pancreatic (712), and gallbladder/biliary (694) (Figure 1; Table 1). The archived primary extraction and the earlier reconstructed sensitivity extraction shared 11,458 operations, with 13 different operations in each. Patient identifiers and 365-day mortality labels agreed for every shared operation. The three-death difference arose from cohort membership, not relabeling of shared records. The earlier reconstruction also used different cancer grouping and predictors; its validation results are replaced by analyses of the original cohort (Supplementary Table 1). Laboratory missingness in INSPIRE is summarized in Supplementary Table 2 and Supplementary Figure 1.

**Figure 1:**
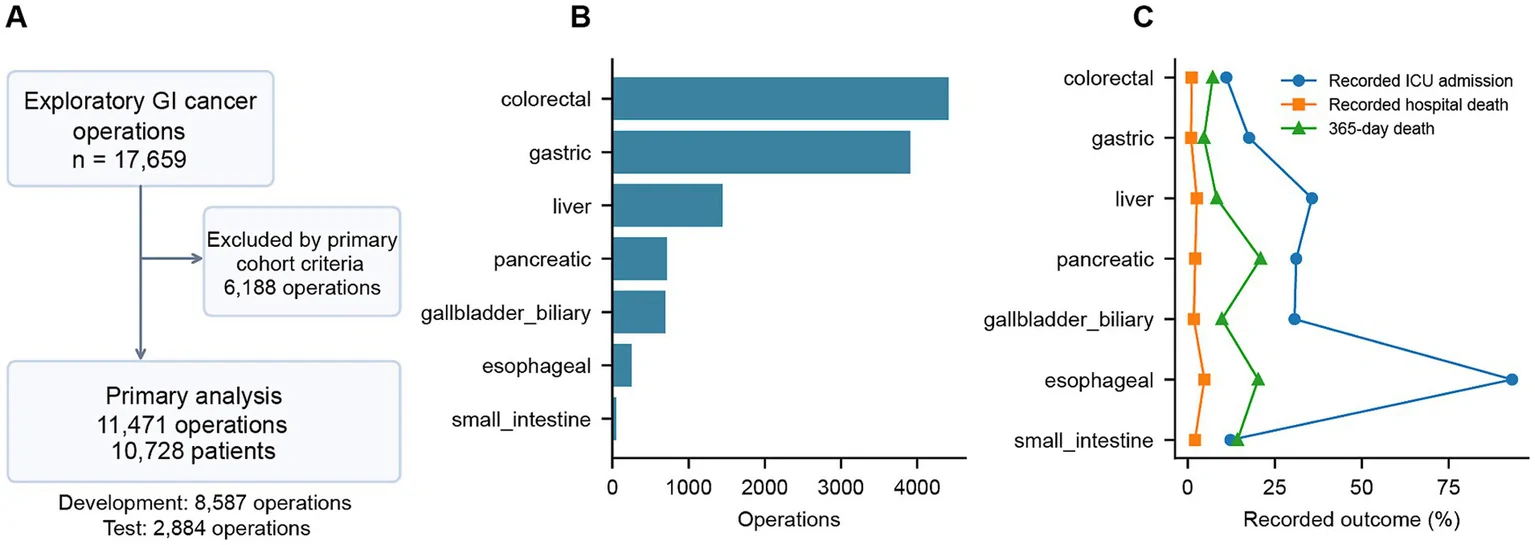
INSPIRE cohort derivation and outcome distribution. **(A)** Flow from exploratory gastrointestinal cancer operations to the primary analytical cohort and unique patients. **(B)** Number of operations by cancer group. **(C)** Observed rates of database-recorded intensive care unit (ICU) admission, recorded hospital death, and 365-day all-cause mortality by cancer group.

**Table 1:** Cohort characteristics and nutritional laboratory summaries by cancer group.

Cancer group	Operations	Patients	Age, median (years)	Female, %	Recorded ICU admission, %	Recorded hospital death, %	365-day mortality, %	Albumin (g/dL)	Hemoglobin (g/dL)
Colorectal	4,413	4,023	65	39.3	11.1	1.1	7.2	4.0	12.5
Gastric	3,906	3,716	60	34.5	17.6	0.9	4.7	4.0	13.2
Liver	1,445	1,356	60	23.4	35.7	2.6	8.3	4.0	13.2
Pancreatic	712	679	65	44.4	31.2	2.1	20.9	4.0	12.4
Gallbladder/ biliary	694	669	70	41.8	30.7	1.7	9.8	3.9	12.5
Esophageal	252	239	65	11.1	93.3	4.8	20.2	4.0	12.9
Small intestine	49	46	55	42.9	12.2	2.0	14.3	4.0	12.2

Albumin and hemoglobin are expressed in g/dL; age in years. ICU, intensive care unit. The colorectal group includes C21 anal malignancies. Secondary outcomes are database-recorded flags, with timing-restricted analyses reported separately.

### Laboratory variables and 365-day mortality discrimination

In the unchanged 2,884-operation test set (233 deaths), the clinical model achieved an AUROC of 0.783 (95% CI 0.752–0.811), AUPRC of 0.289, and Brier score of 0.188. Adding the full laboratory panel yielded an AUROC of 0.843 (95% CI 0.820–0.868), AUPRC of 0.351, and Brier score of 0.159. The full perioperative model achieved an AUROC of 0.856 (95% CI 0.834–0.879), AUPRC of 0.379, and Brier score of 0.156 (Figure 2; Table 2). Adding albumin and hemoglobin alone yielded an AUROC of 0.828, an increment of 0.045 (paired patient-bootstrap 95% CI 0.023–0.067); the remaining laboratory panel added 0.015 (95% CI 0.005–0.026) beyond those two measurements (Supplementary Table 3). Full-pipeline bootstrap-corrected development AUROC was 0.837 (Supplementary Table 4; Supplementary Figure 2). The 7-day, 14-day, missing-indicator, and first-operation sensitivities yielded test AUROCs of 0.852, 0.853, 0.854, and 0.860, respectively. Development-only tuning selected unweighted ridge C = 0.1, with a test AUROC of 0.855 and Brier score of 0.061 (Supplementary Tables 4–6). This lower Brier score reflects calibration as well as discrimination and must not be conflated with the original class-balanced scores.

**Figure 2:**
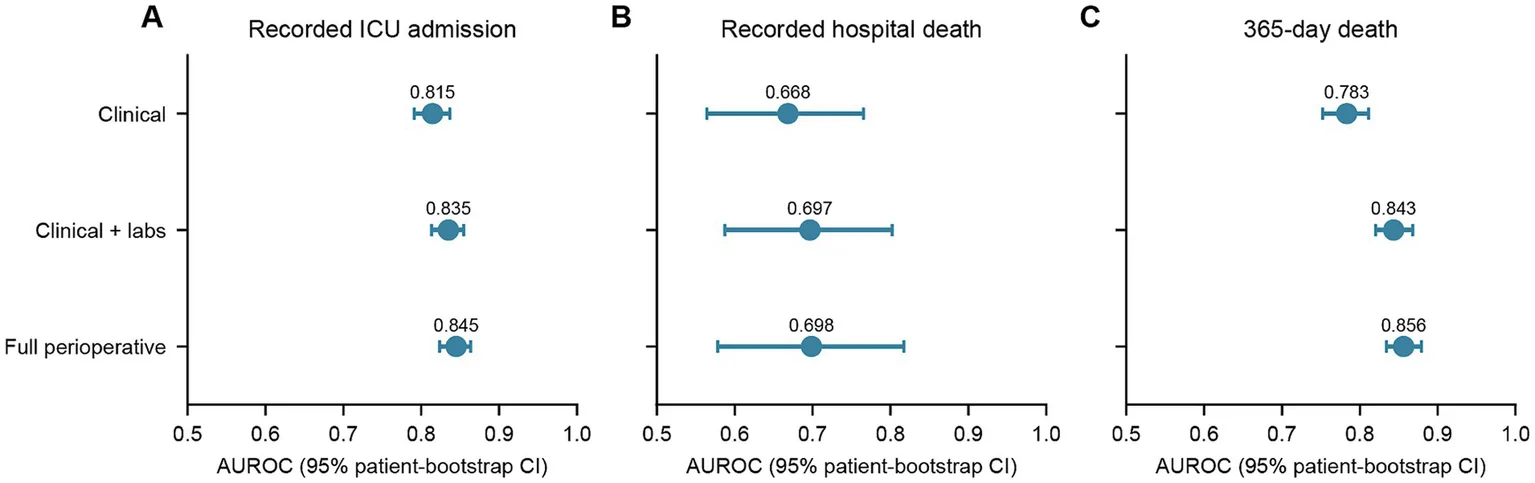
Incremental discrimination of nested models in the INSPIRE test set. Panels show the area under the receiver operating characteristic curve (AUROC) and patient-bootstrap 95% confidence interval for **(A)** recorded ICU admission, **(B)** recorded hospital death, and **(C)** 365-day mortality. “Full perioperative” includes clinical variables, laboratory measurements, and intraoperative summaries.

**Table 2:** Incremental model performance in INSPIRE.

Outcome	Feature set	Events	AUROC	AUROC low	AUROC high	AUPRC	Brier
Recorded ICU	Clinical	629	0.815	0.791	0.837	0.649	0.161
Recorded ICU	Clinical + labs	629	0.835	0.813	0.855	0.680	0.150
Recorded ICU	Full perioperative	629	0.845	0.824	0.863	0.699	0.143
Recorded hospital death	Clinical	39	0.668	0.564	0.765	0.041	0.181
Recorded hospital death	Clinical + labs	39	0.697	0.587	0.802	0.077	0.156
Recorded hospital death	Full perioperative	39	0.698	0.578	0.817	0.083	0.154
365-day mortality	Clinical	233	0.783	0.752	0.811	0.289	0.188
365-day mortality	Clinical + labs	233	0.843	0.820	0.868	0.351	0.159
365-day mortality	Full perioperative	233	0.856	0.834	0.879	0.379	0.156

### Secondary perioperative outcomes and model interpretation

The original recorded-ICU full-model AUROC was 0.845, and the recorded-hospital-death AUROC was 0.698 (Table 2). The source labels were non-missing timestamps. Of 164 hospital-death flags, 95 lay outside the interval from operation end to index discharge; 574 of 2,369 ICU flags lay outside that interval. These observations do not establish that the database labels are erroneous, but they limit their operation-specific interpretation. Timing-restricted sensitivities retained 69 hospital-death events and 1,795 ICU events in the full cohort and are reported separately (Supplementary Table 7). We, therefore, treat secondary endpoint results as exploratory source-record analyses, not validated estimates of strictly postoperative index-admission outcomes. Class balancing caused substantial absolute-risk miscalibration in the original full models (Figure 3). Their decision curves do not establish clinical benefit without a defined intervention and threshold. Figure 4 presents selected fitted coefficients, not causal effects or directly comparable measures of importance (24, 25). The exploratory portable-model ICU comparison is presented in Supplementary Figure 3; its source endpoint definitions differ between cohorts.

**Figure 3:**
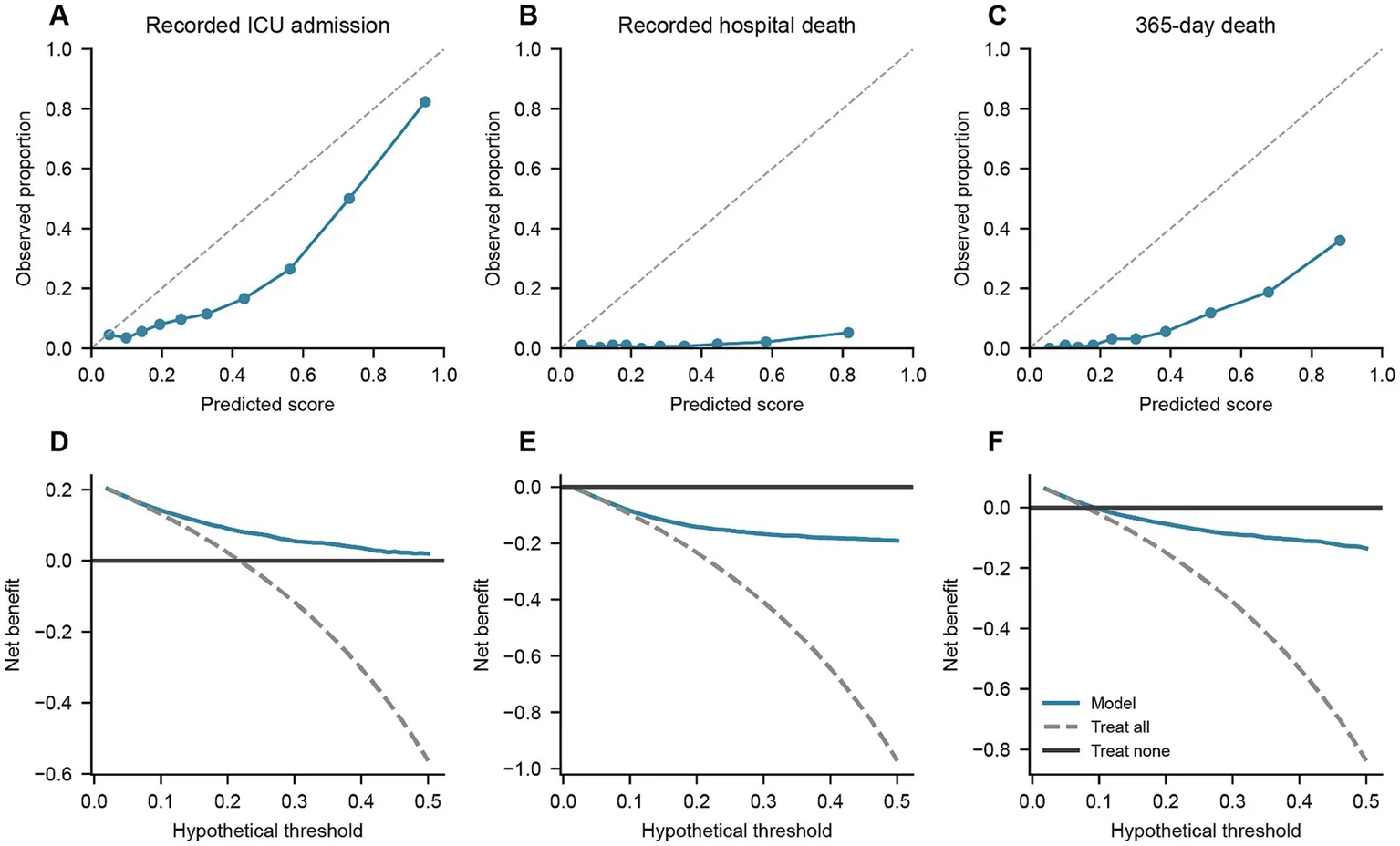
Calibration and exploratory decision-curve analysis of the full perioperative model in the INSPIRE test set. Panels **(A–C)** compare predicted scores and observed event proportions for recorded ICU admission, recorded hospital death, and 365-day mortality; the dashed diagonal denotes equality between predicted and observed values. Panels **(D–F)** show net benefit across hypothetical score thresholds relative to treat-all and treat-none strategies. Class-balanced model scores are not calibrated absolute probabilities; these curves do not establish validated clinical decision rules or clinical benefit.

**Figure 4:**
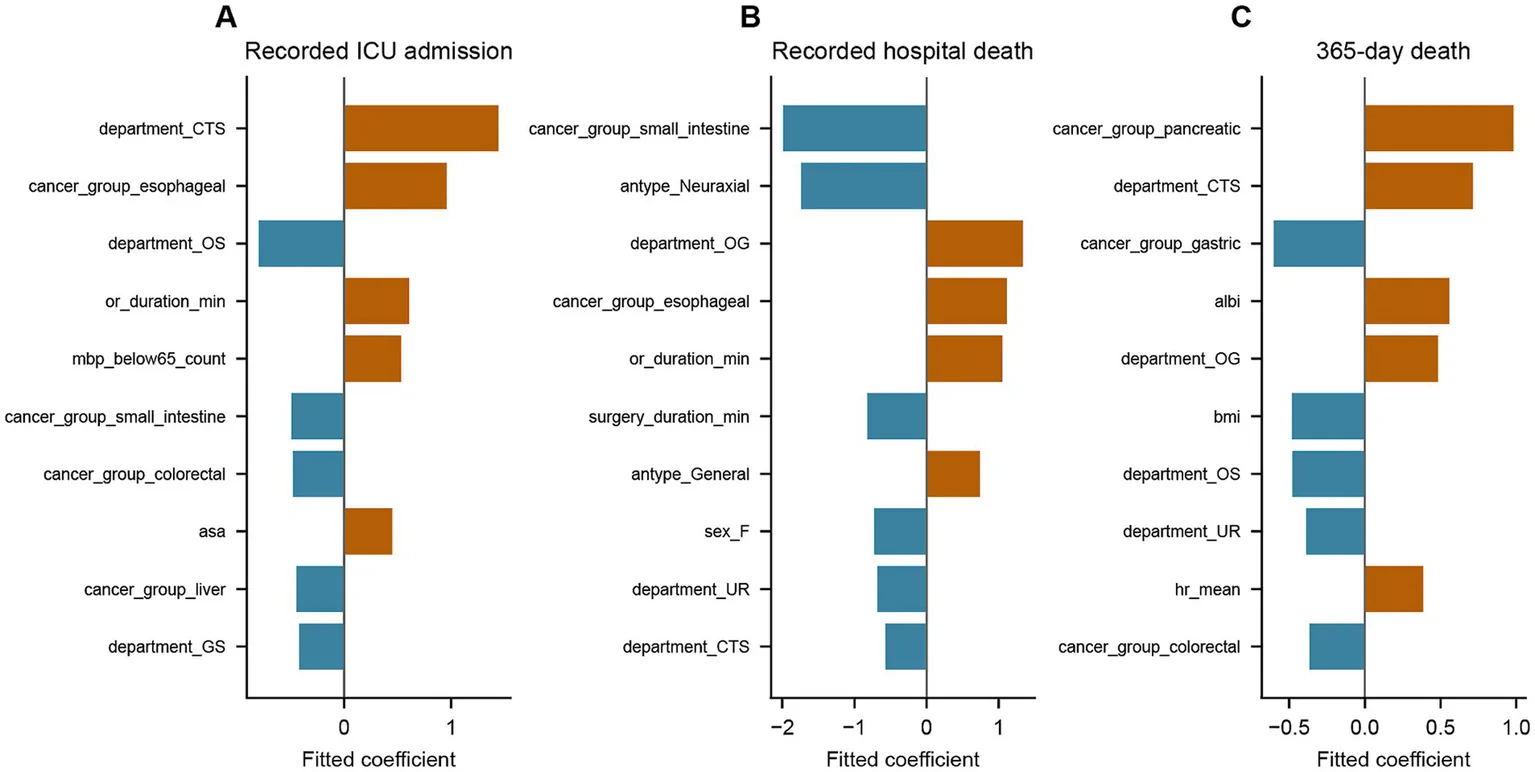
Selected fitted L2-penalized logistic regression coefficients for **(A)** recorded ICU admission, **(B)** recorded hospital death, and **(C)** 365-day mortality. Numeric variables were standardized. All categorical levels were encoded without dropping a reference level; categorical coefficients are contributions in the fitted penalized parameterization, not odds ratios against an omitted reference category. Coefficients are descriptive model parameters, not causal effects or directly comparable importance measures. Department codes: CTS, cardiothoracic surgery; GS, general surgery; OG, obstetrics and gynecology; OS, orthopedic surgery; UR, urology. ALBI, albumin-bilirubin score; BMI, body mass index; ASA, American Society of Anesthesiologists physical status; MBP, mean blood pressure; HR, heart rate. Duration variables are in minutes; mbp_below65_count denotes the count of measurements below 65 mmHg.

### External validation in MIMIC-IV

MIMIC-IV included 8,456 admissions among 5,546 patients, with 3,800 recorded deaths within 365 days and 349 hospital-death flags. After CRP unit harmonization, portable-model AUROC was 0.724 (95% CI 0.692–0.754) for hospital death and 0.676 (95% CI 0.663–0.688) for 365-day mortality (Figure 5; Table 3). For 365-day mortality, the joint calibration intercept was 0.228, the slope was 0.287, calibration-in-the-large was 1.398, and the observed-to-expected ratio was 1.792. For hospital death, the corresponding joint intercept, slope, and observed-to-expected ratio were −2.016, 0.377, and 0.564, respectively. Cross-fitted recalibration reduced the 365-day Brier score from 0.277 to 0.227, without materially improving discrimination. Strictly pre-procedure-date laboratories yielded a 365-day AUROC of 0.675 but a hospital-death AUROC of 0.611, indicating that the latter result was sensitive to laboratory timing. External calibration and recalibration are detailed in Supplementary Table 8, and cross-cohort baseline summaries are provided in Supplementary Table 9. Missingness information is provided in Supplementary Table 2 and Supplementary Data. Exploratory post-recalibration decision-curve results are provided in Supplementary Data and do not establish clinical utility. The MIMIC-IV ICU definition required ICU entry on or after the procedure date and before discharge; it did not capture any ICU stay and could not resolve within-day postoperative timing.

**Figure 5:**
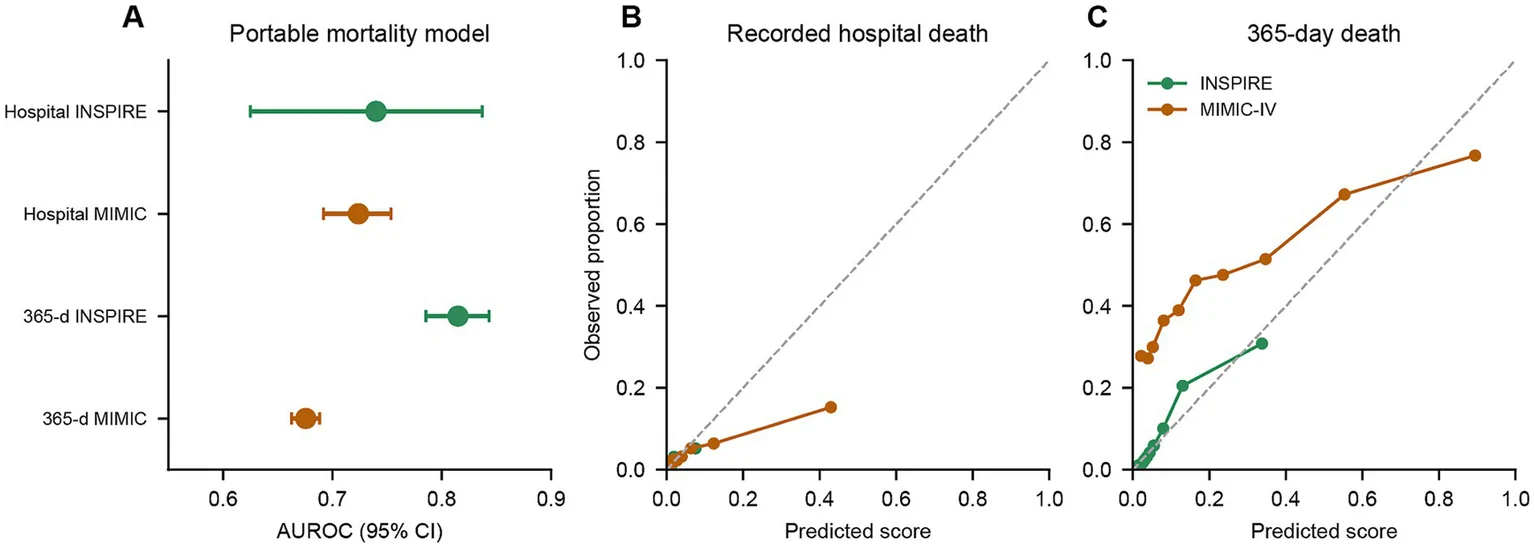
Mortality transportability and calibration of the portable clinical-laboratory model. **(A)** AUROC estimates with patient-bootstrap 95% confidence intervals for recorded hospital death and 365-day mortality in the INSPIRE internal test set and MIMIC-IV external cohort. **(B)** Calibration curves for recorded hospital death. **(C)** Calibration curves for 365-day mortality. Green lines denote INSPIRE internal performance, orange lines denote MIMIC-IV external performance, and the dashed diagonal denotes ideal calibration.

**Table 3:** Portable clinical-laboratory model mortality validation in INSPIRE and MIMIC-IV.

Dataset	Outcome	Events	N	AUROC	AUROC low	AUROC high	AUPRC	Brier
INSPIRE internal	Recorded hospital death	39	2,884	0.740	0.625	0.837	0.108	0.013
MIMIC-IV external	Recorded hospital death	349	8,456	0.724	0.692	0.754	0.142	0.050
INSPIRE internal	365-day mortality	233	2,884	0.815	0.785	0.843	0.313	0.065
MIMIC-IV external	365-day mortality	3,800	8,456	0.676	0.663	0.688	0.630	0.277

### Descriptive laboratory profiles across risk strata

After correcting the admission-level many-to-many merge, INSPIRE risk strata totaled exactly 2,884 operations and 233 deaths: low risk, 15 of 962 (1.56%); intermediate risk, 36 of 961 (3.75%); and high risk, 182 of 961 (18.94%). The corrected high-to-low mortality ratio was 12.15. After CRP unit harmonization, MIMIC-IV mortality ranged from 29.02 to 63.39% across tertiles (Figure 6; Table 4). Higher-risk groups showed lower albumin and hemoglobin; these profiles are descriptive because the same laboratory variables contribute to the risk score. Cancer-specific AUROC varied: 0.867 in colorectal/anal cancer, 0.903 in gastric cancer, 0.779 in liver cancer, and 0.698 in pancreatic cancer. Estimates for smaller cancer groups were imprecise; these analyses do not establish uniform performance across gastrointestinal malignancies (Supplementary Table 10; Supplementary Figure 4).

**Figure 6:**
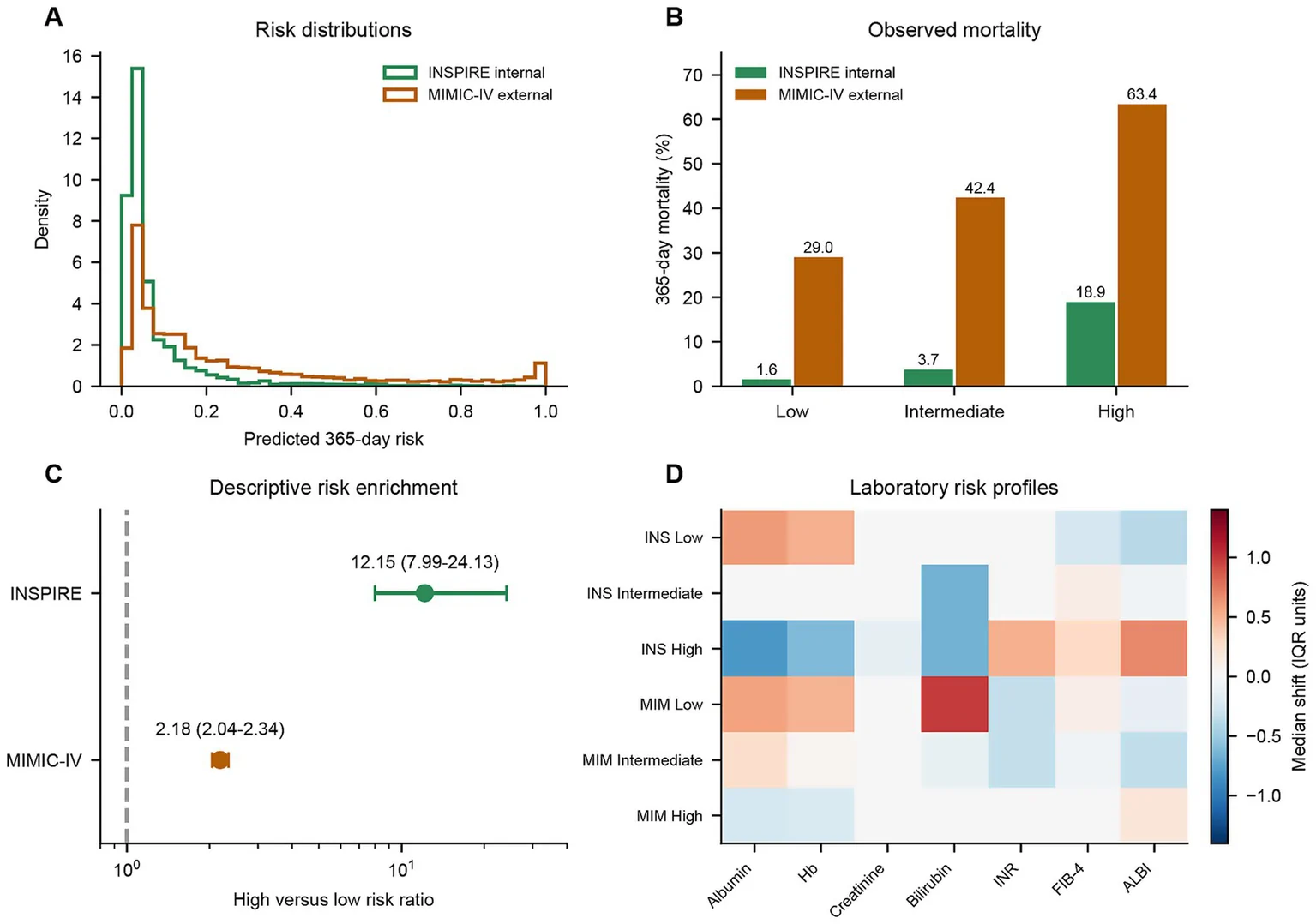
Corrected operation-linked risk strata and laboratory profiles. **(A)** Portable-score distributions. **(B)** Recorded 365-day mortality by cohort-specific tertile. **(C)** High-to-low mortality ratios with 500 patient-level bootstrap percentile intervals, conditional on the fitted scores and tertile definitions. **(D)** Laboratory medians centered and scaled by the corresponding cohort median and interquartile range. The INSPIRE sample comprises 2,884 operations and 233 deaths; the MIMIC-IV sample comprises 8,456 admissions and 3,800 deaths, with CRP units harmonized before scoring.

**Table 4:** Risk-stratified 365-day mortality.

Dataset	Risk stratum	N	Deaths	Observed 365-day mortality, %	Median predicted risk
INSPIRE internal	Low	962	15	1.559	0.022
INSPIRE internal	Intermediate	961	36	3.746	0.040
INSPIRE internal	High	961	182	18.939	0.116
MIMIC-IV external	Low	2,819	818	29.017	0.041
MIMIC-IV external	Intermediate	2,818	1,195	42.406	0.139
MIMIC-IV external	High	2,819	1787	63.391	0.496

## Discussion

Routine laboratory measurements added discriminatory information for 365-day mortality beyond clinical variables in the INSPIRE cohort. Albumin and hemoglobin accounted for part of this increment, while the remaining laboratory panel provided additional information. Similar discrimination across several internal sensitivity analyses supported the stability of this finding within INSPIRE. External performance was more limited, indicating that internal discrimination did not translate into comparable performance across settings.

The contribution of albumin and hemoglobin is consistent with research on nutrition-related vulnerability in gastrointestinal cancer. Studies of nutritional indices, oral frailty, and albumin combined with functional measures provide a clinical context for interpreting these laboratory associations (5–9, 26). The additional information from the wider panel suggests that risk prediction may benefit from considering nutrition-related measurements alongside inflammatory and organ-function indicators. This interpretation concerns prognostic information rather than a specific nutritional mechanism. Our datasets lacked the direct nutritional and functional assessments needed to distinguish nutritional depletion from other sources of laboratory abnormalities. The 2025 ESPEN surgical nutrition guideline considers frailty, sarcopenia, and prehabilitation within perioperative care (27), highlighting dimensions that were not captured here. Nutritional intervention and prehabilitation studies address whether treatment improves outcomes (28); the present prediction analysis does not answer that question. Broader electronic-health-record and multimodal or cancer-specific prediction studies offer complementary approaches (10–16, 29–33). Differences in populations, outcomes, and validation designs prevent direct comparison of their reported AUROCs with those from our study.

External evaluation showed limited 365-day discrimination and substantial calibration drift. Differences in cancer mix, care pathways, laboratory availability, and measurement timing may contribute to the performance gap between INSPIRE and MIMIC-IV. The present analysis cannot isolate the contribution of each source of dataset variation. Cross-fitted recalibration improved the Brier score without materially improving discrimination, indicating that adjustment of absolute risks alone did not resolve limited risk separation. Hospital-death discrimination deteriorated when procedure-day laboratory measurements were excluded. These timing-sensitive results limit interpretation of the portable model as a preoperative prediction tool. Cross-fitted recalibration represents updating within MIMIC-IV, whereas bootstrap analysis and cross-validation in INSPIRE provide internal evaluation rather than additional independent validation.

Several limitations affect interpretation and potential use. Broad procedure codes do not establish curative intent or tumor resection, and the colorectal grouping includes anal malignancies. The original cancer categorization used diagnostic history extending beyond the operation. Preoperative-only assignment changed 124 operation categories and yielded a similar test AUROC of 0.854, but does not establish the main model as a preoperative tool. Tumor stage, histology, neoadjuvant treatment, frailty, sarcopenia, dietary intake, formal nutritional assessment, nutritional intervention, and postoperative complications were not consistently available. Laboratory signals may therefore reflect disease burden or organ dysfunction as well as nutritional vulnerability. Median imputation and missingness indicators cannot eliminate bias from informative missingness. Source outcome timestamps and non-equivalent ICU definitions limit interpretation of secondary endpoints; independent adjudication and complete individual follow-up verification were unavailable. Patient-level splitting, resampling, and a first-operation sensitivity analysis addressed repeated records, although the primary point-estimation model did not include patient random effects. The risk strata remain descriptive because the laboratory measurements used to characterize them also contribute to their defining prediction scores. They do not independently establish biological phenotypes. No prospective intervention, actionable threshold, reclassification rule, or impact evaluation was defined, so clinical benefit from score-guided care remains untested.

In conclusion, nutrition-related laboratory measurements improved discrimination for recorded 365-day mortality beyond clinical variables in INSPIRE. Their incremental prognostic information supports further study of perioperative risk stratification in patients with gastrointestinal cancer. Limited external discrimination, calibration drift, and timing-sensitive secondary outcomes warrant further validation before any clinical use.

## Data Availability

INSPIRE version 1.4.2 was accessed through PhysioNet on June 30, 2026 (10.13026/1eay-yc85) and is described by Lim et al. (17). MIMIC-IV version 3.1 was accessed through PhysioNet on November 20, 2025 (10.13026/kpb9-mt58) and is described by Johnson et al. (18). Access to both datasets is subject to the applicable credentialing and data-use requirements, and the source datasets cannot be redistributed by the authors. Aggregate analytic outputs, numerical specifications of the original-cohort refitted models, a parameter-based scoring utility and refit-verification results are supplied in Supplementary Data. This archive does not contain individual-level data or constitute a complete raw-data extraction and analysis environment.
