# Supplementary material for: Perioperative nutrition-related laboratory profiles predict outcomes after digestive procedures in patients with gastrointestinal cancer
Source: Front Nutr. 2026 Oct 15;13:1927720. doi: 10.3389/fnut.2026.1927720 (PMC13716669; doi:10.3389/fnut.2026.1927720)
Supplement: Supplementary file 1 [file Supplementary_file_1.DOCX]

# Supplementary Table 1 Cohort reconciliation

| **Comparison** | **Operations** | **Patients** | **Deaths** | **Patient ID disagreements** | **Death label disagreements** |
| --- | --- | --- | --- | --- | --- |
| Original primary | 11,471 | 10,728 | 893 | — | — |
| Earlier secondary reconstruction | 11,471 | 10,729 | 890 | — | — |
| Shared records | 11,458 | — | — | 0 | 0 |
| Original only | 13 | — | 3 | — | — |
| Reconstruction only | 13 | — | 0 | — | — |

Deaths refer to 365-day mortality. Disagreement counts apply to shared records. — indicates an unreported or inapplicable field.

# Supplementary Table 2 Missingness of INSPIRE variables

| **Variable** | **Operations** | **Missing n** | **Missing %** |
| --- | --- | --- | --- |
| Age | 11,471 | 0 | 0.00 |
| Sex | 11,471 | 0 | 0.00 |
| ASA physical status | 11,471 | 137 | 1.19 |
| Emergency operation | 11,471 | 0 | 0.00 |
| Body mass index | 11,471 | 157 | 1.37 |
| Albumin | 11,471 | 1,028 | 8.96 |
| Albumin timing (days before operation) | 11,471 | 1,028 | 8.96 |
| Total bilirubin | 11,471 | 1,049 | 9.14 |
| Total bilirubin timing (days before operation) | 11,471 | 1,049 | 9.14 |
| AST | 11,471 | 1,037 | 9.04 |
| AST timing (days before operation) | 11,471 | 1,037 | 9.04 |
| ALT | 11,471 | 1,041 | 9.08 |
| ALT timing (days before operation) | 11,471 | 1,041 | 9.08 |
| ALP | 11,471 | 1,042 | 9.08 |
| ALP timing (days before operation) | 11,471 | 1,042 | 9.08 |
| INR | 11,471 | 1,364 | 11.89 |
| INR timing (days before operation) | 11,471 | 1,364 | 11.89 |
| Platelet count | 11,471 | 1,028 | 8.96 |
| Platelet count timing (days before operation) | 11,471 | 1,028 | 8.96 |
| CRP | 11,471 | 5,576 | 48.61 |
| CRP timing (days before operation) | 11,471 | 5,576 | 48.61 |
| White blood cell count | 11,471 | 1,009 | 8.80 |
| White blood cell count timing (days before operation) | 11,471 | 1,009 | 8.80 |
| Hemoglobin | 11,471 | 1,010 | 8.80 |
| Hemoglobin timing (days before operation) | 11,471 | 1,010 | 8.80 |
| Creatinine | 11,471 | 859 | 7.49 |
| Creatinine timing (days before operation) | 11,471 | 859 | 7.49 |
| Lactate | 11,471 | 7,004 | 61.06 |
| Lactate timing (days before operation) | 11,471 | 7,004 | 61.06 |
| Glucose | 11,471 | 1,233 | 10.75 |
| Glucose timing (days before operation) | 11,471 | 1,233 | 10.75 |
| HbA1c | 11,471 | 9,546 | 83.22 |
| HbA1c timing (days before operation) | 11,471 | 9,546 | 83.22 |
| Blood urea nitrogen | 11,471 | 1,062 | 9.26 |
| Blood urea nitrogen timing (days before operation) | 11,471 | 1,062 | 9.26 |
| Sodium | 11,471 | 230 | 2.01 |
| Sodium timing (days before operation) | 11,471 | 230 | 2.01 |
| Potassium | 11,471 | 231 | 2.01 |
| Potassium timing (days before operation) | 11,471 | 231 | 2.01 |
| Number of available laboratory tests | 11,471 | 0 | 0.00 |
| ALBI score | 11,471 | 1,049 | 9.14 |
| Albumin-to-bilirubin ratio | 11,471 | 1,049 | 9.14 |
| APRI | 11,471 | 1,130 | 9.85 |
| FIB-4 | 11,471 | 1,134 | 9.89 |

Missingness is reported before imputation. Laboratory timing fields are listed separately from laboratory measurements. Exact MIMIC-IV missing counts, denominators and percentages are provided in Supplementary Data/MIMIC_missingness.csv; Supplementary Table 9 also reports observed sample sizes. ASA, American Society of Anesthesiologists; ALBI, albumin-bilirubin; APRI, AST-to-platelet ratio index; FIB-4, fibrosis-4 index; AST, aspartate aminotransferase; ALT, alanine aminotransferase; ALP, alkaline phosphatase; INR, international normalized ratio; CRP, C-reactive protein; HbA1c, glycated hemoglobin.

# Supplementary Table 3 Incremental performance of laboratory predictors

| **Comparison** | **ΔAUROC (95% CI)** | **ΔAUPRC (95% CI)** | **ΔBrier (95% CI)** |
| --- | --- | --- | --- |
| Clinical + albumin + hemoglobin versus clinical | 0.045 (0.023 to 0.067) | 0.048 (0.009 to 0.082) | -0.018 (-0.022 to -0.014) |
| Clinical + all laboratories versus clinical + albumin + hemoglobin | 0.015 (0.005 to 0.026) | 0.014 (-0.020 to 0.050) | -0.011 (-0.014 to -0.008) |
| Clinical + all laboratories versus clinical | 0.060 (0.035 to 0.084) | 0.062 (0.017 to 0.112) | -0.029 (-0.034 to -0.024) |

Differences are the first model minus the comparator. Positive AUROC/AUPRC differences and negative Brier differences favor the first model. AUROC, area under the receiver operating characteristic curve; AUPRC, area under the precision-recall curve; CI, confidence interval. Parentheses following performance estimates contain 95% CIs.

# Supplementary Table 4 Full-pipeline bootstrap internal validation

| **Metric** | **Apparent** | **Mean optimism** | **Optimism-corrected** |
| --- | --- | --- | --- |
| AUROC | 0.851 | 0.015 | 0.837 |
| AUPRC | 0.348 | 0.025 | 0.323 |
| Brier score | 0.156 | -0.002 | 0.158 |

Internal validation used 500 patient-level bootstrap samples and repeated the full modeling pipeline. Corrected performance equals apparent performance minus mean optimism. AUROC, area under the receiver operating characteristic curve; AUPRC, area under the precision-recall curve.

# Supplementary Table 5 Development-selected unweighted ridge model

| **Model characteristic or metric** | **Value** |
| --- | --- |
| Inverse regularization strength C | 0.100 |
| Operations | 2,884 |
| Patients | 2,682 |
| Events | 233 |
| AUROC | 0.855 (0.834 to 0.880) |
| AUPRC | 0.392 (0.331 to 0.453) |
| Brier score | 0.061 (0.054 to 0.068) |
| Calibration intercept | 0.056 |
| Calibration slope | 1.025 |
| Calibration-in-the-large | 0.009 |
| Observed/expected ratio | 1.007 |
| Mean cross-validation AUROC | 0.832 |
| Mean cross-validation log loss | 0.216 |

Analysis: development-selected unweighted ridge logistic regression. The regularization parameter was selected in the development cohort. Test-set performance is reported for 365-day mortality. AUROC, area under the receiver operating characteristic curve; AUPRC, area under the precision-recall curve; CI, confidence interval. Parentheses following performance estimates contain 95% CIs. Calibration intercept and slope are jointly estimated. Calibration-in-the-large is the intercept estimated with the prediction slope fixed at 1. Observed/expected is the ratio of observed to expected events.

# Supplementary Table 6 Sensitivity analyses for predictor availability and model specification

| **Analysis** | **Records** | **Patients** | **Events** | **AUROC (95% CI)** | **AUPRC (95% CI)** | **Brier (95% CI)** |
| --- | --- | --- | --- | --- | --- | --- |
| 7-day laboratory window | 2,884 | 2,682 | 233 | 0.852 (0.828 to 0.875) | 0.377 (0.317 to 0.441) | 0.158 (0.149 to 0.165) |
| 14-day laboratory window | 2,884 | 2,682 | 233 | 0.853 (0.830 to 0.877) | 0.377 (0.318 to 0.440) | 0.157 (0.149 to 0.165) |
| Missingness indicators | 2,884 | 2,682 | 233 | 0.854 (0.830 to 0.878) | 0.392 (0.329 to 0.461) | 0.151 (0.143 to 0.160) |
| Unweighted L2 model | 2,884 | 2,682 | 233 | 0.854 (0.833 to 0.877) | 0.393 (0.332 to 0.455) | 0.061 (0.054 to 0.068) |
| First operation only | 2,682 | 2,682 | 191 | 0.860 (0.839 to 0.884) | 0.359 (0.298 to 0.424) | 0.157 (0.148 to 0.165) |
| Clinical + albumin + hemoglobin | 2,884 | 2,682 | 233 | 0.828 (0.803 to 0.853) | 0.337 (0.278 to 0.394) | 0.170 (0.162 to 0.178) |

## Calibration

| **Analysis** | **Intercept** | **Slope** | **Calibration-in-the-large** | **Observed/expected** |
| --- | --- | --- | --- | --- |
| 7-day laboratory window | -2.430 | 0.882 | -2.504 | 0.230 |
| 14-day laboratory window | -2.436 | 0.866 | -2.525 | 0.231 |
| Missingness indicators | -2.404 | 0.846 | -2.514 | 0.242 |
| Unweighted L2 model | 0.016 | 0.996 | 0.024 | 1.018 |
| First operation only | -2.587 | 0.859 | -2.692 | 0.206 |
| Clinical + albumin + hemoglobin | -2.433 | 0.927 | -2.474 | 0.218 |

AUROC, area under the receiver operating characteristic curve; AUPRC, area under the precision-recall curve; CI, confidence interval. Parentheses following performance estimates contain 95% CIs. Calibration intercept and slope are jointly estimated. Calibration-in-the-large is the intercept estimated with the prediction slope fixed at 1. Observed/expected is the ratio of observed to expected events.

Records are INSPIRE operations.

# Supplementary Table 7 Timing-restricted secondary outcomes

| **Analysis** | **Records** | **Patients** | **Events** | **AUROC (95% CI)** | **AUPRC (95% CI)** | **Brier (95% CI)** |
| --- | --- | --- | --- | --- | --- | --- |
| In-hospital death within index interval | 2,884 | 2,682 | 20 | 0.907 (0.852 to 0.953) | 0.140 (0.057 to 0.320) | 0.088 (0.080 to 0.096) |
| ICU admission within postoperative index interval | 2,884 | 2,682 | 467 | 0.909 (0.895 to 0.922) | 0.715 (0.672 to 0.753) | 0.114 (0.107 to 0.121) |

## Calibration

| **Analysis** | **Intercept** | **Slope** | **Calibration-in-the-large** | **Observed/expected** |
| --- | --- | --- | --- | --- |
| In-hospital death within index interval | -4.650 | 0.425 | -6.689 | 0.042 |
| ICU admission within postoperative index interval | -1.554 | 0.947 | -1.571 | 0.506 |

## Event counts in the full INSPIRE cohort

| **Outcome** | **Timing-restricted events** | **Original non-null events** |
| --- | --- | --- |
| In-hospital death within index interval | 69 | 164 |
| ICU admission within postoperative index interval | 1,795 | 2,369 |

AUROC, area under the receiver operating characteristic curve; AUPRC, area under the precision-recall curve; CI, confidence interval. Parentheses following performance estimates contain 95% CIs. Calibration intercept and slope are jointly estimated. Calibration-in-the-large is the intercept estimated with the prediction slope fixed at 1. Observed/expected is the ratio of observed to expected events.

Records are INSPIRE operations.

# Supplementary Table 8 External performance and cross-fitted recalibration

| **Analysis** | **Records** | **Patients** | **Events** | **AUROC (95% CI)** | **AUPRC (95% CI)** | **Brier (95% CI)** |
| --- | --- | --- | --- | --- | --- | --- |
| 365-day death / External validation | 8,456 | 5,546 | 3,800 | 0.676 (0.663 to 0.688) | 0.630 (0.610 to 0.652) | 0.277 (0.268 to 0.286) |
| 365-day death / Cross-fitted recalibration | 8,456 | 5,546 | 3,800 | 0.676 (0.662 to 0.688) | 0.630 (0.611 to 0.652) | 0.227 (0.224 to 0.231) |
| In-hospital death / External validation | 8,456 | 5,546 | 349 | 0.724 (0.692 to 0.754) | 0.142 (0.117 to 0.175) | 0.050 (0.046 to 0.054) |
| In-hospital death / Cross-fitted recalibration | 8,456 | 5,546 | 349 | 0.723 (0.690 to 0.753) | 0.140 (0.115 to 0.174) | 0.038 (0.034 to 0.042) |
| ICU admission / External validation | 8,456 | 5,546 | 1,477 | 0.589 (0.572 to 0.605) | 0.234 (0.217 to 0.251) | 0.290 (0.282 to 0.298) |
| ICU admission / Cross-fitted recalibration | 8,456 | 5,546 | 1,477 | 0.589 (0.572 to 0.605) | 0.233 (0.217 to 0.250) | 0.142 (0.138 to 0.147) |

## Calibration

| **Analysis** | **Intercept** | **Slope** | **Calibration-in-the-large** | **Observed/expected** |
| --- | --- | --- | --- | --- |
| 365-day death / External validation | 0.228 | 0.287 | 1.398 | 1.792 |
| 365-day death / Cross-fitted recalibration | -0.001 | 0.995 | 0.000 | 1.000 |
| In-hospital death / External validation | -2.016 | 0.377 | -0.882 | 0.564 |
| In-hospital death / Cross-fitted recalibration | -0.015 | 0.995 | 0.001 | 1.001 |
| ICU admission / External validation | -1.555 | 0.115 | -2.383 | 0.394 |
| ICU admission / Cross-fitted recalibration | -0.021 | 0.986 | -0.000 | 1.000 |

AUROC, area under the receiver operating characteristic curve; AUPRC, area under the precision-recall curve; CI, confidence interval. Parentheses following performance estimates contain 95% CIs. Calibration intercept and slope are jointly estimated. Calibration-in-the-large is the intercept estimated with the prediction slope fixed at 1. Observed/expected is the ratio of observed to expected events.

Records are MIMIC-IV admissions. Cross-fitted recalibration is model updating, not independent validation. ICU admission is an exploratory cross-cohort comparison.

# Supplementary Table 9 Baseline characteristics across cohorts

| **Characteristic** | **INSPIRE observed n** | **INSPIRE median (IQR) or %** | **MIMIC-IV observed n** | **MIMIC-IV median (IQR) or %** |
| --- | --- | --- | --- | --- |
| Age (years) | 11,471 | 65.00 (55.00–70.00) | 8,456 | 67.00 (58.00–76.00) |
| Albumin (g/dL) | 10,443 | 4.00 (3.70–4.20) | 3,022 | 3.10 (2.70–3.50) |
| Total bilirubin (mg/dL) | 10,422 | 0.70 (0.50–0.80) | 4,410 | 1.30 (0.60–4.40) |
| AST (IU/L) | 10,434 | 21.00 (17.00–28.00) | 4,409 | 61.00 (27.00–138.00) |
| ALT (IU/L) | 10,430 | 18.00 (13.00–27.00) | 4,391 | 48.00 (21.00–124.00) |
| ALP (IU/L) | 10,429 | 71.00 (55.00–86.00) | 4,394 | 172.00 (90.00–397.75) |
| INR (dimensionless) | 10,107 | 1.00 (0.95–1.05) | 5,182 | 1.30 (1.10–1.40) |
| Platelet count (10⁹/L) | 10,443 | 233.00 (178.00–273.00) | 6,282 | 209.00 (143.00–291.00) |
| CRP (mg/dL) | 5,895 | 0.19 (0.06–0.77) | 117 | 5.62 (1.48–11.96) |
| White blood cell count (10⁹/L) | 10,462 | 5.94 (4.77–7.45) | 6,282 | 8.40 (5.80–12.10) |
| Hemoglobin (g/dL) | 10,461 | 12.90 (11.30–14.20) | 6,286 | 10.30 (8.90–11.78) |
| Creatinine (mg/dL) | 10,612 | 0.80 (0.68–0.98) | 6,381 | 0.80 (0.60–1.10) |
| Lactate (mmol/L) | 4,467 | 0.90 (0.80–1.20) | 2,578 | 1.70 (1.20–2.60) |
| Glucose (mg/dL) | 10,238 | 101.00 (91.00–120.00) | 6,329 | 119.00 (97.00–157.00) |
| HbA1c (%) | 1,925 | 6.10 (5.70–7.10) | 164 | 6.40 (5.67–8.30) |
| Blood urea nitrogen (mg/dL) | 10,409 | 13.00 (10.00–18.00) | 6,358 | 15.00 (10.00–21.00) |
| Sodium (mmol/L) | 11,241 | 139.00 (136.00–140.00) | 6,656 | 138.00 (136.00–141.00) |
| Potassium (mmol/L) | 11,240 | 3.80 (3.50–4.20) | 6,934 | 4.00 (3.70–4.40) |
| Male | 7,395 | 64.47% | 5,131 | 60.68% |
| Female | 4,076 | 35.53% | 3,325 | 39.32% |
| Colorectal | 4,413 | 38.47% | 1,983 | 23.45% |
| Gastric | 3,906 | 34.05% | 773 | 9.14% |
| Liver | 1,445 | 12.60% | 1,933 | 22.86% |
| Pancreatic | 712 | 6.21% | 2,359 | 27.90% |
| Gallbladder/biliary | 694 | 6.05% | 759 | 8.98% |
| Esophageal | 252 | 2.20% | 505 | 5.97% |
| Small intestine | 49 | 0.43% | 144 | 1.70% |

INSPIRE: 11,471 operations; MIMIC-IV: 8,456 admissions. For continuous variables, observed n is the number with available measurements and values are median (interquartile range). For categorical variables, observed n is the category count and percentages use all cohort records as the denominator. Units are shown in row labels. For cell counts, /nL and K/µL are equivalent to 10⁹/L; for sodium and potassium, mEq/L is numerically equivalent to mmol/L. MIMIC-IV CRP was converted from mg/L to mg/dL. AST, aspartate aminotransferase; ALT, alanine aminotransferase; ALP, alkaline phosphatase; INR, international normalized ratio; CRP, C-reactive protein; HbA1c, glycated hemoglobin.

# Supplementary Table 10 Performance across cancer and clinical subgroups Discrimination and overall error

| **Subgroup** | **N** | **Patients** | **Events** | **AUROC (95% CI)** | **AUPRC (95% CI)** | **Brier (95% CI)** |
| --- | --- | --- | --- | --- | --- | --- |
| Cancer: Colorectal | 1,159 | 1,046 | 94 | 0.867 (0.826 to 0.906) | 0.436 (0.337 to 0.543) | 0.145 (0.135 to 0.156) |
| Cancer: Esophageal * | 57 | 55 | 11 | 0.733 (0.529 to 0.924) | 0.490 (0.254 to 0.794) | 0.386 (0.313 to 0.446) |
| Cancer: Gallbladder/biliary * | 161 | 155 | 14 | 0.807 (0.671 to 0.913) | 0.402 (0.162 to 0.661) | 0.258 (0.219 to 0.299) |
| Cancer: Gastric | 982 | 932 | 43 | 0.903 (0.859 to 0.943) | 0.365 (0.257 to 0.528) | 0.087 (0.079 to 0.096) |
| Cancer: Liver | 339 | 315 | 28 | 0.779 (0.704 to 0.857) | 0.338 (0.197 to 0.528) | 0.197 (0.172 to 0.224) |
| Cancer: Pancreatic | 165 | 160 | 39 | 0.698 (0.618 to 0.794) | 0.473 (0.340 to 0.632) | 0.374 (0.331 to 0.418) |
| Cancer: Small intestine * | 21 | 19 | 4 | 0.515 | 0.243 | 0.221 |
| Female | 1,029 | 961 | 83 | 0.833 (0.788 to 0.879) | 0.332 (0.251 to 0.448) | 0.153 (0.140 to 0.166) |
| Male | 1,855 | 1,721 | 150 | 0.869 (0.844 to 0.893) | 0.409 (0.335 to 0.492) | 0.158 (0.150 to 0.169) |
| Age ≥65 years | 1,451 | 1,370 | 147 | 0.829 (0.793 to 0.858) | 0.391 (0.312 to 0.465) | 0.204 (0.193 to 0.217) |
| Age <65 years | 1,433 | 1,322 | 86 | 0.882 (0.849 to 0.916) | 0.366 (0.286 to 0.468) | 0.108 (0.098 to 0.116) |
| Elective | 2,668 | 2,514 | 186 | 0.848 (0.820 to 0.874) | 0.316 (0.257 to 0.393) | 0.145 (0.138 to 0.152) |
| Emergency | 216 | 213 | 47 | 0.830 (0.770 to 0.884) | 0.594 (0.432 to 0.725) | 0.302 (0.264 to 0.348) |
| ASA III or higher | 277 | 264 | 49 | 0.824 (0.766 to 0.884) | 0.564 (0.422 to 0.694) | 0.301 (0.262 to 0.336) |
| ASA I–II | 2,573 | 2,429 | 176 | 0.850 (0.823 to 0.880) | 0.306 (0.249 to 0.387) | 0.140 (0.131 to 0.147) |
| Missing ASA * | 34 | 34 | 8 | 0.865 (0.725 to 0.979) | 0.660 (0.325 to 0.933) | 0.228 (0.141 to 0.335) |
| Hepatobiliary or pancreatic | 665 | 630 | 81 | 0.788 (0.739 to 0.830) | 0.400 (0.303 to 0.501) | 0.256 (0.239 to 0.276) |
| Other cancer groups | 2,219 | 2,052 | 152 | 0.874 (0.843 to 0.902) | 0.399 (0.314 to 0.478) | 0.126 (0.119 to 0.136) |

N denotes operations. * Fewer than 20 events; interpret cautiously. Confidence intervals are not reported for subgroups with fewer than five events. AUROC, area under the receiver operating characteristic curve; AUPRC, area under the precision-recall curve; CI, confidence interval. Parentheses following performance estimates contain 95% CIs.

# Supplementary Table 10 Performance across cancer and clinical subgroups Calibration

| **Subgroup** | **Intercept** | **Slope** | **Calibration-in-the-large** | **Observed/expected** |
| --- | --- | --- | --- | --- |
| Cancer: Colorectal | -2.382 | 1.050 | -2.356 | 0.239 |
| Cancer: Esophageal * | -2.678 | 1.093 | -2.555 | 0.283 |
| Cancer: Gallbladder/biliary * | -2.857 | 0.764 | -3.149 | 0.178 |
| Cancer: Gastric | -2.443 | 1.155 | -2.431 | 0.182 |
| Cancer: Liver | -2.500 | 0.670 | -2.790 | 0.210 |
| Cancer: Pancreatic | -1.850 | 0.485 | -2.741 | 0.346 |
| Cancer: Small intestine * | -1.439 | 0.008 | -0.867 | 0.597 |
| Female | -2.313 | 0.786 | -2.427 | 0.241 |
| Male | -2.515 | 0.916 | -2.580 | 0.229 |
| Age ≥65 years | -2.459 | 0.824 | -2.621 | 0.239 |
| Age <65 years | -2.352 | 0.955 | -2.364 | 0.224 |
| Elective | -2.429 | 0.900 | -2.475 | 0.214 |
| Emergency | -2.354 | 0.764 | -2.813 | 0.359 |
| ASA III or higher | -2.535 | 0.834 | -2.804 | 0.301 |
| ASA I–II | -2.421 | 0.884 | -2.474 | 0.215 |
| Missing ASA * | -1.981 | 0.861 | -2.164 | 0.434 |
| Hepatobiliary or pancreatic | -2.399 | 0.654 | -2.846 | 0.250 |
| Other cancer groups | -2.392 | 1.053 | -2.370 | 0.225 |

* Fewer than 20 events; interpret cautiously. Calibration intercept and slope are jointly estimated. Calibration-in-the-large is the intercept estimated with the prediction slope fixed at 1. Observed/expected is the ratio of observed to expected events. ASA, American Society of Anesthesiologists. Outcomes are 365-day deaths in the INSPIRE test set.

## Supplementary methods and figure legends

## Cohort definition and reconciliation

The INSPIRE primary rule was diagnosis at or before opstart_time, an operation code beginning 0D/0F, and exclusion of the other_digestive category. Cancer priority was gastric, colorectal/anal, liver, pancreatic, gallbladder/biliary, esophageal, small intestine, then unspecified digestive. It did not require a particular department, nonnegative opdate, or resection-only root. The earlier reconstruction excluded 11 original operations through its department restriction, one through a negative opdate restriction, and one through use of opdate rather than operation start for diagnosis timing; it included 13 C26-group operations excluded by the primary rule. The original-only operations contained three 365-day deaths; the reconstruction-only operations contained none. The shared 11,458 operations had identical patient identifiers and death labels. The 4,117 differing cancer-group labels in the shared records reflected different mapping rules, including erroneous grouping of C18/C19 in the secondary reconstruction, not changes to the primary patient data. That non-equivalent reconstruction and its 195-column validation model are superseded by the original-cohort analyses.

## Predictor transformations and reproducible specification

ALBI = 0.66 log10(total bilirubin [mg/dL] x 17.104) - 0.085 albumin [g/dL] x 10. APRI = (AST/40)/platelets x 100. FIB-4 = age x AST/(platelets x sqrt(ALT)). The albumin-bilirubin ratio used both measurements in their original g/dL and mg/dL scales. Converted bilirubin (µmol/L) and ALT were bounded below at 0.001 within the logarithm and square root, respectively; zero denominators produced missing values. Infinite derived values were set missing. BMI outside 10-80 was missing. Other laboratory features were not log-transformed. The primary clinical/full pipelines used L2 C=1, balanced class weights, liblinear, max_iter=3000, training-only median imputation and standardization. Categorical missingness was literal missing and all categories were included. The portable model omitted class weights. Numeric imputation medians, scaling means and standard deviations, category order, intercepts, and coefficients from original-cohort refits are provided in Supplementary Data/Model specifications. The file refit_verification.csv quantifies numerical differences from archived original predictions. The accompanying scoring utility applies these exported parameters to prepared feature tables; it does not recreate raw-data extraction or cross-fitted recalibration. See README.txt for input conventions and limitations.

## Resampling and sensitivity analyses

The original patient split used GroupShuffleSplit, seed 20260702, with 8,046 development patients and 2,682 test patients. Patient-bootstrap intervals resample patient clusters with replacement and preserve all their operations/admissions; they condition on the fitted scores and do not include training uncertainty. Full-pipeline optimism resampling separately refits preprocessing and the model in 500 development-patient bootstrap samples and evaluates each on the bootstrap and original development samples. Mean optimism is subtracted from apparent performance. This procedure is not independent validation. First-operation analysis retained the earliest eligible operation per patient and the original patient allocation. For 7-day and 14-day windows, values outside the narrower window were set missing and derived indices recomputed before training-only imputation. The missing-indicator sensitivity appended indicators for numeric features missing in training. No multiple imputation was performed. Unweighted ridge C was selected from 0.01, 0.1, 1, and 10 by five-fold patient-grouped mean validation log loss within development; all preprocessing was refitted within folds. These additional analyses were specified during revision, not prospectively preregistered. Evaluation intervals use 500 patient-bootstrap samples; subgroup intervals use 300 samples when at least five events are available.

## External timing and calibration

MIMIC-IV laboratory item mappings were checked against the source dictionary. CRP and high-sensitivity CRP were combined as in the original extraction; their source unit was mg/L and was divided by 10 to match INSPIRE mg/dL. Primary corrected external estimates use the original measurements and cohort with that unit correction. The separate preprocedure-date sensitivity re-extracted the most recent same-admission measurement strictly before midnight starting the procedure date, within 30 days, requiring storetime before that date when available. This is conservative because actual procedure times are unavailable. Calibration-in-the-large uses an offset logistic intercept with slope fixed at one; the joint intercept and slope fit outcome against predicted log odds. Observed-to-expected is the observed event count divided by the sum of predicted probabilities. Cross-fitted recalibration fits intercept and slope in four patient folds and evaluates in the fifth. The resulting evaluation concerns updating within MIMIC-IV, not independent validation. Decision curves use hypothetical thresholds and establish no intervention benefit.

## Secondary endpoint uncertainty

Original INSPIRE ICU/hospital-death labels were defined by nonmissing source timestamps. Timing-restricted analyses require event time between operation end and index discharge; this rule yielded 1,795 ICU flags and 69 hospital-death flags. Neither definition was independently clinically adjudicated. The MIMIC-IV ICU rule was ICU entry on or after the qualifying procedure date and no later than discharge, not any ICU admission. Exact within-day postoperative status cannot be verified. These secondary endpoints and their differing eligibility/timing must not be used as evidence of harmonized postoperative prediction.

## Risk groups and subgroup interpretation

INSPIRE portable predictions were originally saved with admission identifiers. Joining these to operation rows by subject and admission produced 40 extra rows. The correction verified original split order, patient identifiers, admission identifiers, and outcomes before assigning operation identifiers, yielding one row per test operation. Tertiles were recalculated on 2,884 operations. MIMIC-IV tertiles were recalculated after unit harmonization on 8,456 admissions. Conditional patient-bootstrap risk-ratio intervals use fixed fitted scores and group definitions. Subgroup estimates report all specified cancer groups, sex, age, emergency status, ASA class, and hepatopancreatobiliary versus other sites. Confidence intervals are omitted for groups with fewer than five events and estimates with fewer than 20 events are flagged as sparse. No subgroup is claimed to have equivalently validated performance, and these exploratory comparisons were not used to select the model. The hepatobiliary/pancreatic grouping uses liver, pancreatic and gallbladder/biliary cancer sites, not a verified major-resection or procedure-complexity classification.

# Supplementary figure legends

Supplementary Figure 1. Missing proportions for the 17 source laboratory predictors in the primary INSPIRE cohort. The denominator is the original cohort of 11,471 operations.

Supplementary Figure 2. Original-cohort internal validation. (A) Distribution of AUROC optimism across 500 patient-level full-pipeline bootstrap refits. (B) Apparent development, optimism-corrected development, and fixed held-out AUROC. The bootstrap analysis estimates optimism in discrimination; it does not estimate a corrected calibration curve.

Supplementary Figure 3. Exploratory portable-model ICU comparison. (A) Patient-bootstrap AUROC intervals. (B) Calibration by score decile. INSPIRE nonmissing ICU timestamps and MIMIC-IV date-based ICU entry are non-equivalent endpoints; this is not harmonized postoperative ICU validation.

Supplementary Figure 4. Cancer-specific test AUROC for recorded 365-day death, with patient-bootstrap 95% intervals where at least five events were observed. Labels give operations and events. Sparse groups do not establish reliable site-specific performance.
